# Intensive care unit-acquired infections more common in patients with COVID-19 than with influenza

**DOI:** 10.1038/s41598-024-67733-z

**Published:** 2024-07-19

**Authors:** Josefine Beck-Friis, Magnus Gisslén, Staffan Nilsson, Anna Lindblom, Jonatan Oras, Aylin Yilmaz

**Affiliations:** 1 Department of Infectious Diseases, Institute of Biomedicine, Sahlgrenska Academy, University of Gothenburg, Gothenburg, Sweden; 2 Department of Infectious Diseases, Region Västra Götaland, Sahlgrenska University Hospital, 416 85 Gothenburg, Sweden; 3 Public Health Agency of Sweden, Solna, Sweden; 4 Department of Laboratory Medicine, Institute of Biomedicine, Sahlgrenska Academy, University of Gothenburg, Gothenburg, Sweden; 5 Department of Clinical Microbiology, Region Västra Götaland, Sahlgrenska University Hospital, Gothenburg, Sweden; 6 Department of Anesthesiology and Intensive Care Medicine, Institute of Clinical Sciences, Sahlgrenska Academy, University of Gothenburg, Gothenburg, Sweden; 7 Department of Anesthesia and Intensive Care, Region Västra Götaland, Sahlgrenska University Hospital, Gothenburg, Sweden

**Keywords:** SARS-CoV-2, Influenza, Ventilator-associated pneumonia, Glucocorticoids, Bacteria, Mortality, Clinical microbiology, Bacterial infection, Viral infection, Respiratory distress syndrome

## Abstract

Intensive care unit-acquired infections are complicating events in critically ill patients. In this study we analyzed the incidence, microbiological patterns, and outcome in patients with COVID-19 versus influenza in the intensive care unit (ICU). We included all adult patients treated with invasive mechanical ventilation due to (1) COVID-19 between January 2020 and March 2022, and (2) influenza between January 2015 and May 2023 at Sahlgrenska University Hospital, Sweden. Of the 480 participants included in the final analysis, 436 had COVID-19. The incidence rates of ICU-acquired infections were 31.6/1000 and 9.9/1000 ICU-days in the COVID-19 and influenza cohorts, respectively. Ventilator-associated lower respiratory tract infections were most common in both groups. In patients with COVID-19, corticosteroid treatment was associated with an increased risk of ICU-acquired infections and with higher 90-day mortality in case of infection. Furthermore, ICU-acquired infection was associated with a prolonged time in the ICU, with more difficult-to-treat gram-negative infections in late versus early ventilator-associated lower respiratory tract infections. Further research is needed to understand how the association between corticosteroid treatment and incidence and outcome of ICU-acquired infections varies across different patient categories.

## Introduction

Patients with viral pneumonia in the intensive care unit (ICU) are at risk of secondary infections that may result in greater length of stay (LoS) and higher morbidity and mortality^1–3^. Intensive care unit-acquired infections (ICU-AI) have been reported in 15–25% of patients admitted to the ICU, with ventilator-associated pneumonia (VAP) being the most common infection among patients requiring invasive mechanical ventilation (IMV)^4,5^. Pre-pandemic studies of VAP have reported the highest prevalence in patients with prior trauma, chronic obstructive pulmonary disease, and acute respiratory distress syndrome (ARDS)^6,7^. The association between VAP and increased LoS in ICUs and time on IMV is well established, but the correlation with mortality remains controversial^8^.

During the COVID-19 pandemic, approximately 14% of patients hospitalized due to COVID-19 were admitted to intensive care, mainly because of ARDS^9^. Compared to influenza, patients with COVID-19 had longer ICU LoS and duration of IMV, as well as higher mortality^10,11^. In June 2020, dexamethasone 6 mg once daily was introduced worldwide as the standard of care for patients with COVID-19-related hypoxia, following positive results on mortality and duration of hospitalization from the RECOVERY trial^12^. Concerns were soon raised that glucocorticoids could increase the risk of bacterial and fungal infections, although studies showed conflicting results^13,14^. The association between ICU-AI, glucocorticoids, and ICU outcome remains uncertain^2,15,16^. Furthermore, the potential differences between ICU-AI in patients with COVID-19 and those with influenza have not been thoroughly explored. In this study, we compared the incidence, microbial patterns, and outcomes of ICU-AI in patients with COVID-19 versus influenza. We also performed an in-depth analysis of the COVID-19 cohort to determine the association between ICU-AI and corticosteroid treatment.

## Methods

### Study design and participants

We conducted a retrospective observational cohort study at Sahlgrenska University Hospital (SU) in Gothenburg, Sweden, including all patients 18 years and older on IMV with an International Classification of Diseases 10th Revision (ICD-10) code for COVID-19 between February 2020 and March 2022 (Supplementary Table 1). Patients with an ICD-10 code for influenza were included from January 2015 to May 2023. Exclusion criteria were IMV < 48 h, transfer to/from an ICU outside of SU, or main diagnosis other than either COVID-19 or influenza. Waves of the pandemic were determined by each increase in the number of hospitalized patients with COVID-19 at SU (Wave 1: 1 Feb 2020–27 Sep 2020, Wave 2: 28 Sep 2020–31 Jan 2021, Wave 3: 1 Feb 2021–7 Nov 2021, and Wave 4: 8 Nov 2021–31 Mar 2022; Supplementary Fig. 1). All research was performed in accordance with the Declaration of Helsinki and relevant guidelines. The study protocol was reviewed and approved by the Swedish Ethical Review Authority (IRB number registration number 2020-01771 and 2022-00653-02), which waived the requirement for informed consent due to the observational nature of the study.

### Data collection

Data regarding patient demographics, co-infections, simplified acute physiology score III (SAPS 3)^17^, ICU LoS, days on IMV, immunomodulatory and antimicrobial treatment, clinical and biochemical signs of infection (C-reactive protein and white blood cell count) as well as 30- and 90-day mortality were collected from medical charts. A Charlson Comorbidity Index score (CCI) was calculated based on comorbidities recorded in the medical charts^18^. From the microbiology laboratory at SU, we collected results from blood and lower respiratory tract cultures, polymerase chain reaction (PCR) testing of samples from the lower respiratory tract, and urine antigen tests (*Streptococcus pneumoniae* and *Legionella pneumophila*) from hospital admission and up to 48 h after discharge from the ICU.

### Classification of infections and microbiological findings

Definitions according to the European Centre for Disease Prevention and Control were used for healthcare-associated infections and significance of microbiological findings^19^. Multidrug-resistant organisms (MDROs) were classified according to an international expert proposal^20^ and discussed with a specialist consultant in microbiology (AL) as needed. Several isolates in the same sample were considered as multiple infections, while repeated cultures with the same isolate were considered a single infection, unless there had been a clear clinical improvement and at least seven days between cultures. *Candida* spp in respiratory samples were considered as colonization. The term ventilator-associated lower respiratory tract infection (VA-LRTI) was used instead of VAP due to difficulties with interpreting radiological findings in patients with COVID-19. Considering the generally long ICU LoS among patients with COVID-19, the cut-off between early and late ICU-AI was defined by the median number of days until the first ICU-AI in the COVID-19 cohort, instead of the more common cut-off at five days. Cases that were difficult to define according to the set definitions were discussed among the co-authors.

#### ICU-AI

Infection diagnosed ≥ two days after admittance to the ICU or ≤ two days after discharge. Only infections confirmed by microbiological findings and clinical symptoms were included.

#### VA-LRTI

Presence of at least one of the following during invasive mechanical ventilation: (a) fever > 38 °C or (b) leukopenia (< 4000 WBC/mm^3^) or leukocytosis (> 12, 000 WBC/mm^3^) *and* at least two of the following: (c) new onset of or change in purulent sputum, (d) cough or dyspnea or tachypnea, (e) suggestive auscultation, or (f) worsening gas exchange *and* one of the following microbiological findings: (g) positive quantitative culture from bronchoalveolar lavage (BAL), protected-brush, or endotracheal aspirate, (h) positive sputum or non-quantitative lower respiratory tract specimen culture, or (i) alternative microbiological tests (PCR test, urine antigen test for *Legionella pneumophila* or *Streptococcus pneumoniae*).

#### Blood stream infection (BSI)

One positive blood culture of a recognized pathogen or a combination of clinical symptoms (fever > 38 °C, chills, and/or hypotension) *and* two positive blood cultures of a common skin contaminant from two separate blood samples drawn within 48 h.

#### Co-infection

Bacterial infection diagnosed with clinical signs and microbiological findings < 48 h after admittance to the ICU.

### Statistical analysis

Descriptive data were reported as frequencies and percentages for categorical variables and as median and ranges for continuous variables. For comparison between groups, Fisher’s exact test was used for categorical data and the Mann–Whitney U test for continuous variables. ANOVA, Pearson’s chi-squared, and Kruskal–Wallis tests were used as appropriate for comparisons between three or more groups. A *P*-value < 0.05 was considered significant. Ventilator-free day was defined as the number of days the patient was alive and free of mechanical ventilation after being intubated. We set the time frame at 28 days, thus giving the patient a value of 0 if they died before day 28 or were still receiving mechanical ventilation at day 28. The incidence rates of first ICU-AI were calculated by dividing the number of cases with their first ICU-AI with days at risk (all days in the ICU for patients with no ICU-AI, added to all days in the ICU until the first ICU-AI for the remaining patients) × 1000 days. For patients for whom the date of ICU-AI was missing, the days at risk were calculated as ICU LoS divided by two. For incidence rates of first VA-LRTI, the days at risk consisted of all days on IMV for patients without VA-LRTI added to all days on IMV until the first VA-LRTI for the remaining patients. Poisson 95% confidence intervals (CI) were calculated for incidence rates and compared using chi-square statistic.

The cumulative incidence of ICU-AI with and without corticosteroid treatment was calculated and displayed using a Fine-Gray model, considering discharge from ICU or death as competing events. Sub-hazard ratios were calculated using the same model, adjusting for confounders (age, sex, immunosuppressive treatment at baseline, SAPS 3, and CCI score) in order to identify factors associated with ICU-AI. Hazard ratios for 90-day mortality, adjusted for age, sex, SAPS 3, and CCI score, were calculated using Cox regression with ICU-AI as a time-dependent covariate. Analysis and graphical figures were computed using Microsoft Excel version 16.77, IBM SPSS Statistics version 29.0.0.0, R version 4.2.2, Affinity Designer 2 version 2.2.0, and GraphPad Prism version 10.0.3.

## Results

### Study population

**Figure 1:**
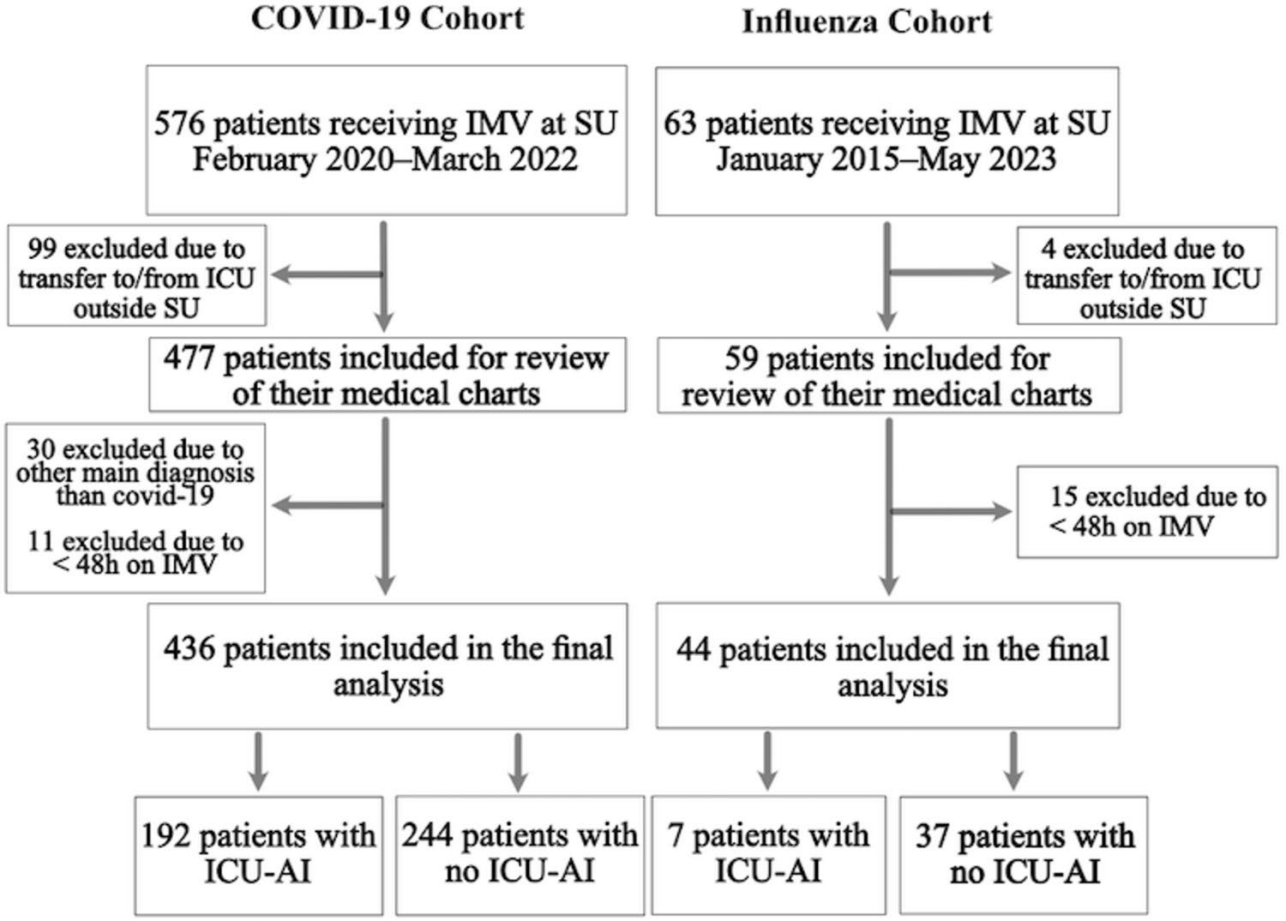
Flowchart showing the number of included and excluded patients in the study. Patients were stratified into two major cohorts (COVID-19 and Influenza) and two subgroups in each major cohort (ICU-AI and no ICU-AI). *ICU* Intensive care unit, *ICU-AI* ICU-Acquired Infection, *IMV* Invasive mechanical ventilation, *SU* Sahlgrenska University Hospital.

We identified 576 patients with COVID-19 on IMV in five different ICUs at SU during the study period (Fig. 1). After exclusion of patients who did not fulfill the inclusion criteria, 436 individuals remained in the COVID-19 cohort. Of these, 160 were admitted in Wave one, 112 in Wave two, 144 in Wave three, and 20 in Wave four. In the final analysis, 44 patients with influenza were included in the comparison group, of which 31 (70%) had influenza A. Five cases with influenza occurred towards the end of or after the pandemic (2022–2023), and the rest before the COVID-19 pandemic in Sweden. Median age in both cohorts was 63 years (range 20–90; Table 1). The groups were comparable with regard to comorbidities and previous immunosuppressive therapy. There were more males than females in the COVID-19 cohort. Patients with influenza had more severe illness at the time of admission with a higher SAPS 3 and more co-infections.

**Table 1:** Baseline Characteristics of Patients on Invasive Mechanical Ventilation.

	COVID-19 (N = 436)	Influenza (N = 44)
Median (range) or n (%)		
Sex		
Female	107 (25)	19 (43)
Age in years	63 (20–90)	63 (29–86)
Charlson Comorbidity Index score	3 (0–9)	2 (0–7)
Number of comorbidities	2 (0–9)	2 (0–7)
Hypertension	208 (48)	21 (48)
Diabetes	125 (29)	7 (16)
Heart disease^a^	62 (14)	6 (14)
COPD	17 (4)	10 (24)
Immunosuppressive therapy	30 (7)	3 (7)
Corticosteroids^b^	13 (3)	2 (5)
Cytotoxic chemotherapy	10 (2)	1 (2)
Other	16 (4)^c^	2 (5)^d^
Co-infection at admission^e^	49 (11)	16 (36)
SAPS 3	51 (34–108)^f^	60 (40–93)^g^

*COPD* Chronic obstructive pulmonary disease, *SAPS 3* Simplified Acute Physiology Score 3 to predict hospital mortality on ICU admission.

Definitions: Co-infection = bacterial infection diagnosed < 48 h after admittance to Intensive Care Unit (ICU) due to SARS CoV-2 or influenza virus infection.

^a^Heart diseases include congestive heart failure, ischemic heart disease, and previous myocardial infarction.

^b^Prednisolon in doses 5–15 mg OD.

^c^3 monoclonal antibodies, 7 tacrolimus, 1 ruxolitinib, 6 mycophenolic acid, 4 ciclosporin, 1 abatacept, 2 everolimus, 1 azathioprine, 1 TNF inhibitor.

^d^1 cytarabine, 1 tacrolimus, 1 mycophenolic acid.

^e^Hospital-acquired infection and co-infection at hospitalization are both included here. Hospital-acquired infection represent N = 15 (COVID-19) and N = 2 (influenza).

^f^N = 433 due to missing values.

^g^N = 40 due to missing values.

### Incidence of ICU-AI

At least one ICU-AI occurred in 192 patients (44%) with COVID-19 and in 7 (16%) with influenza (*P* < 0.001; Table 2). Incidence rates of first ICU-AI/1000 ICU-days were 31.6 (95% CI 27.3–36.4) and 9.9 (95% CI 4.0–20.5) for COVID-19 and influenza, respectively (*P* = 0.002). The difference in incidence rates remained similar when comparing only patients without corticosteroid treatment (22.3 vs 5.6, *P* = 0.026). Incidence rates of first VA-LRTI/1000 ventilator-days were 25.7 (95% CI 21.7–30.1) for COVID-19 and 8.3 (95% CI 2.7–19.4) for influenza (*P* = 0.009). The incidence in the COVID-19 cohort increased in subsequent waves in comparison to the first wave (Supplementary Table 2). There was no significant difference in the incidence of ICU-AI among patients with influenza before and after the COVID-19 pandemic (20% vs 15%, *P* = 0.75). BSI occurred in 77 patients (18%) with COVID-19 and in 3 (7%) with influenza.

**Table 2:** Treatment and Outcome in the Intensive Care Unit.

	COVID-19 (N = 436)	Influenza (N = 44)	*P* value
Median (range) or n (%)			
ICU-acquired infection	192 (44)	7 (16)	< 0.001
Days at ICU until first ICU-AI	9 (2–56)^a^	7 (7–48)	0.86
Incidence rate ICU-AI per 1000 ICU-days (95% CI)	31.6 (27.2–36.4)^b^	9.9 (4.0–20.5)	0.002
Ventilator-associated lower respiratory tract infection	149 (34)	5 (11)	0.002
Days on IMV until first VA-LRTI	8 (1–56)	7 (3–48)	0.63
Incidence rate VA-LRTI per 1000 ventilator days (95% CI)	25.7 (21.7–30.1)	8.3 (2.7–19.4)	0.009
Bloodstream infection	77 (18)	3 (7)	0.087
Other ICU-AI^c^	18 (4)	0	0.39
Days in ICU	18 (2–103)	19 (3–74)	0.13
Days on IMV	15 (2–92)	13 (3–68)	0.039
Ventilator-free days at 28 days	9 (0–27)	14 (0–25)	0.024
ECMO	2 (0.5)	1 (2)	0.24
Anti-inflammatory medicine^d^	299 (69)	24 (55)	0.062
Anti-viral treatment^e^	35 (8)	42 (95)	< 0.001
Antibiotic treatment	434 (100)	44 (100)	1.00
Antibiotic treatment within 48 h	399 (92)	42 (95)	0.56
First antibiotic administered			
Cefotaxime	345 (80)	16 (36)	< 0.001
Piperacillin/tazobactam	70 (16)	16 (36)	0.002
Meropenem	12 (3)	8 (18)	< 0.001
Number of antibiotic drugs	3 (0–14)	3 (1–15)	0.37
Antimycotic treatment	123 (28)	15 (34)	0.48
30-day mortality	102 (23)	8 (18)	0.57
90-day mortality	130 (30)	9 (20)	0.23

*ECMO* Extracorporeal membrane oxygenation, *ICU* Intensive care unit, *ICU-AI* ICU-Acquired Infection, *IMV* Invasive mechanical ventilation, *VA-LRTI* Ventilator-associated lower respiratory tract infection, *95% CI* Poisson 95% confidence interval.

^a^N = 187 due to five patients missing data on days in ICU until first ICU-AI.

^b^Times at risk for the five patients with data missing were estimated to half their lengths of stay in ICU.

^c^Four *Clostridium difficile* enterocolitis, seven with positive culture in lower respiratory tract and fever as single clinical sign, two skin/wound infection, two Herpes simplex infection, one urosepsis, one Cytomegalovirus reactivation.

^d^Anti-inflammatory medicine given in hospital due to viral infection. N = 477 due to data missing in the COVID-19 cohort. In the COVID-19 cohort corticosteroids (298), IL-6 blockers (23), and JAK inhibitors (2) were used, while only corticosteroids were used in the influenza cohort.

^e^Anti-viral treatment given to patients with (1) COVID-19 was remdesivir and (2) influenza was oseltamivir.

### Treatment and outcome

There were no significant differences in mortality or ICU LoS between the COVID-19 and influenza cohorts (Table 2). Median number of days until first ICU-AI was 9 (range 2–56) in the COVID-19 cohort and 7 (range 7–48) in the influenza cohort (*P* = 0.86). VA-LRTI occurred within five days of admission the ICU in 29 patients (19%) with COVID-19 and one (20%) with influenza. The first dose of antibiotics was given within 48 h of ICU admission.

to 92% of patients with COVID-19 and to 95% with influenza. For patients in the COVID-19 cohort, this number was similar throughout all four waves (Supplementary Table 2).

Cefotaxime was the first antibiotic administered to 80% of patients with COVID-19, whereas 16% received piperacillin/tazobactam. The ratio between the administration of cefotaxime and piperacillin/tazobactam decreased throughout the pandemic. In patients with influenza, 36% received cefotaxime and piperacillin/tazobactam respectively, and 18% meropenem. Erythromycin was co-administered to 48% of the patients with influenza, but only to 1% of patients with COVID-19.

Anti-inflammatory treatment, predominantly corticosteroids, was given to 299 patients (69%) with COVID-19 and 24 (55%) with influenza (Table 2). In the first wave, corticosteroids were given to 21% of patients with COVID-19, and to > 90% in subsequent waves. The majority of the patients with COVID-19 received corticosteroids according to Swedish recommendations (betamethasone 6 mg once daily for 10 days) but a few patients may have been given a prolonged treatment. The type of corticosteroids, dose, and duration of treatment varied greatly among patients with influenza. While the indication for corticosteroids was respiratory failure and/or ARDS in only four patients in this cohort, the main indication was airway obstruction (14/24) and in three cases septic shock.

### ICU-AI in the COVID-19 cohort

**Figure 2:**
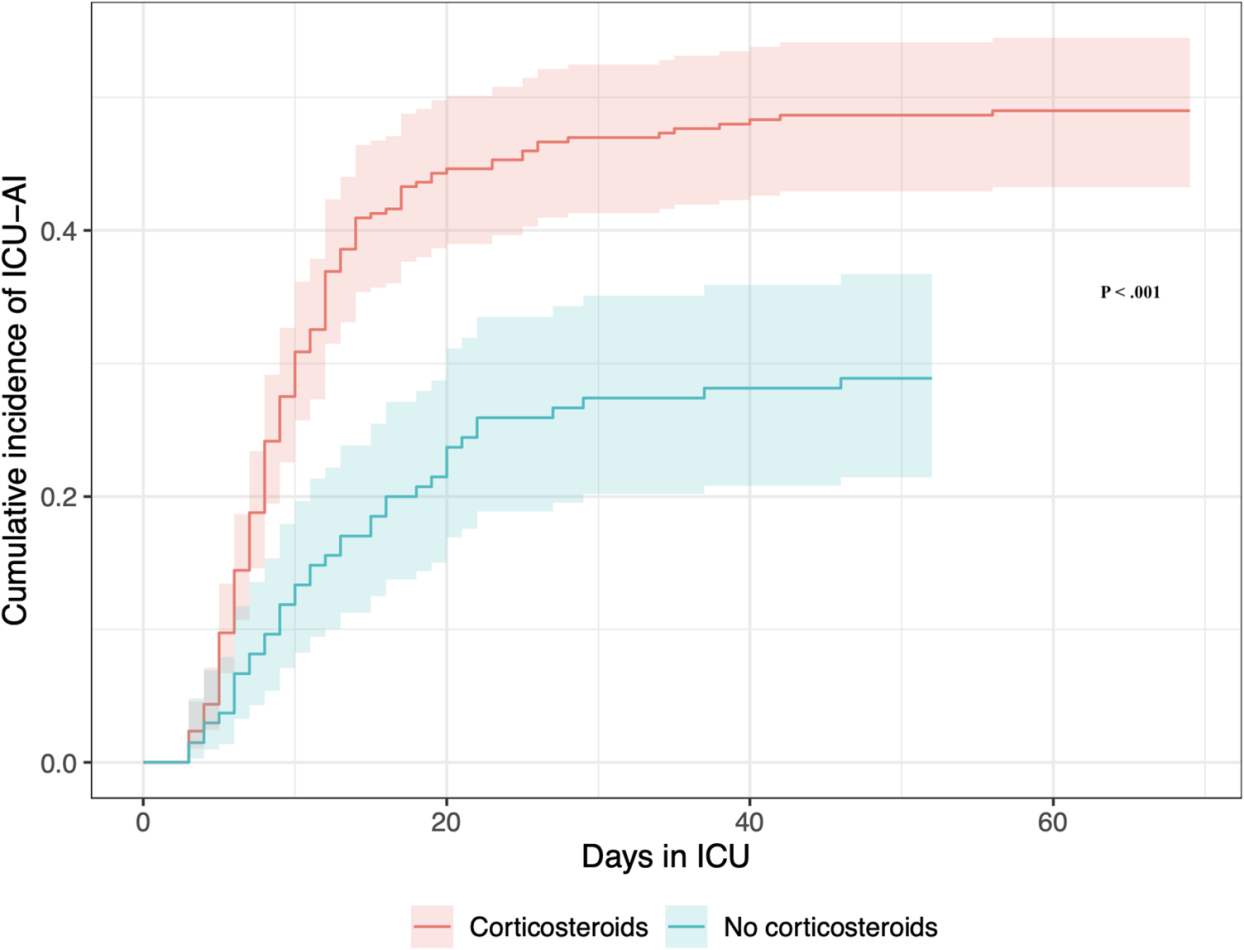
Cumulative incidence of intensive care unit-acquired infections in patients with and without corticosteroid treatment from a competing events analysis using Fine and Gray model with discharge from intensive care or death as competing events. *P* < 0.001. *ICU-AI* ICU-Acquired Infection.

Patients with ICU-AI remained in the ICU for a median 15 days longer (*P* < 0.001) and had a higher 90-day mortality (*P* = 0.045; Table 3). When considering ICU-AI as a time-dependent variable and stratifying for corticosteroid treatment, the adjusted hazard ratios for 90-day mortality were 1.81 (95% CI 1.16–2.84) for patients with corticosteroid treatment, and 0.68 (95% CI 0.33–1.37) for patients without corticosteroids. The cumulative incidence of ICU-AI was higher in the group with corticosteroid treatment in a competing event analysis (Fig. 2). Adjusted sub-hazard ratios for ICU-AI were 2.18 (95% CI 1.54–3.09; *P* < 0.001) with corticosteroid treatment, and 1.72 (95% CI 1.18–2.56; *P* = 0.006) for male gender. There was no significant difference in the median age when comparing patients with or without ICU-AI, and with or without corticosteroids (*P* = 0.008). A comparison of patients within the influenza cohort confirmed the longer ICU LoS and time on IMV in case of ICU-AI (Supplementary Table 3).

**Table 3:** Subgroup Analysis of Patients on Invasive Mechanical Ventilation due to COVID-19.

	Total (N = 436)	ICU-AI (N = 192)	No ICU-AI (N = 244)	*P* value
Median (IQR) or n (%)				
Female	107 (25)	33 (17)	74 (30)	0.002
Age in years	63 (20–90)	64 (31–90)	63 (20–86)	0.201
Charlson Comorbidity Index score	3 (0–9)	3 (0–8)	2 (0–9)	0.36
Number of comorbidities	2 (0–6)	2 (0–6)	1 (0–5)	0.084
Immunosuppressive therapy baseline	31 (7)	9 (5)	22 (9)	0.093
SAPS 3	51 (34–108)	51 (34–100)	51 (34–108)	0.90
Anti-inflammatory medicine at ICU^a^	299 (69)	149 (78)	150 (61)	< 0.001
Corticosteroid treatment	298 (69)	149 (78)	149 (61)	< 0.001
IL-6 receptor blocker	23 (5)	9 (5)	14 (6)	0.83
JAK inhibitor	2 (0.7)	1 (0.5)	2 (0.8)	1.00
Number of antibiotic drugs	3 (0–14)	4.5 (1–14)	2 (0–11)	< 0.001
Days in ICU	18 (2–103)	27 (4–103)	12 (2–69)	< 0.001
Days on IMV	15 (1–92)	24 (3–92)	10 (2–67)	< 0.001
30-day mortality	102 (23)	42 (22)	60 (25)	0.57
90-day mortality	130 (30)	67 (35)	63 (26)	0.045

*ICU* Intensive care unit, *ICU-AI* ICU-acquired infection, *IL-6* Interleukin 6, *IMV* Invasive mechanical ventilation, *JAK* Janus Kinase, *SAPS 3* Simplified Acute Physiology Score 3 to predict hospital mortality on ICU admission.

^a^N = 433 with N = 189 in the ICU-AI group and N = 244 in the no ICU-AI group due to data missing.

### Microbiological findings

**Figure 3:**
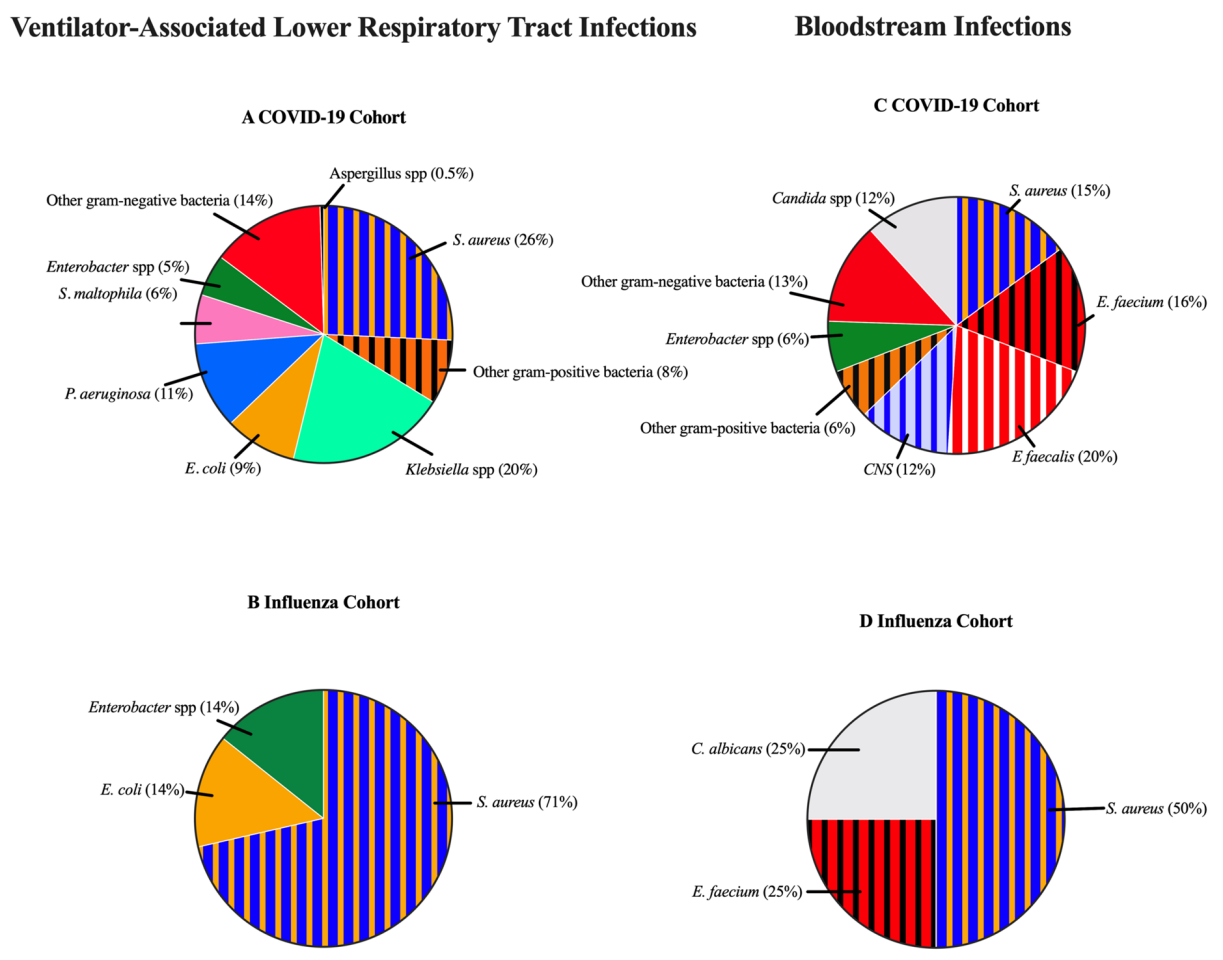
Microbiological findings in intensive care unit-acquired infections. (**A**,**B**) Microbes associated with ventilator-associated lower respiratory tract infections in the COVID-19 (**A**) and influenza (**B**) cohorts. (**C**,**D**) Microbes associated with bloodstream infections in the COVID-19 (**C**) and influenza (**D**) cohorts. Each microbe was only counted once per patient and infection and presented as number (n) and percentage (%). Gram-positive bacteria are marked with stripes. All were culture positive, none of the microbes were identified through alternative methods (PCR-test or urine-antigen test) only. *C. albicans Candida albicans*, *E. coli Escherichia coli, E. faecium Enterococcus faecium, E. faecalis Enterococcus faecalis, P. aeruginosa Pseudomonas aeruginosa, S. aureus Staphylococcus aureus, S. maltophila Stenotrophomonas maltophila, spp* species.

The majority of VA-LRTI (N = 138, 66%) in the COVID-19 cohort were caused by gram-negative bacteria, compared to 28% (N = 2) for patients with influenza (Fig. 3)*.* In the COVID-19 cohort, gram-negative bacteria were more common in late compared to early VA-LRTI (75% versus 56%; Supplementary Table 4). The most notable increases between early and late infection were seen in *Pseudomonas aeruginosa* (7% to 15%) and *Stenotrophomonas maltophila* (1% to 11%).

Sixty-five BSIs in the COVID-19 group were caused by gram-positive bacteria, 18 by gram-negative bacteria, and 11 by *Candida* spp. All BSIs in the influenza cohort were caused by either gram-positive bacteria (3 of 4) or *Candida albicans* (1 of 4). Of all patients with an ICU-AI in the COVID-19 cohort, 28 (15%) had an MDRO, of which 22 (79%) were gram-negative bacteria. None of the infections in the influenza group were caused by an MDRO. (All the blood and lower respiratory tract cultures reviewed in the study are presented in Supplementary Table 5).

## Discussion

In this Swedish retrospective cohort study, mechanically ventilated patients with COVID-19 experienced a higher incidence of ICU-acquired infections compared to those with influenza. *Staphylococcus aureus* was identified as the most common pathogen causing VA-LRTI among patients with influenza and COVID-19, while gram-negative bacteria as a group caused the majority of VA-LRTI in patients with COVID-19. We found an association between ICU-AI and increased risk of mortality in patients treated with corticosteroids. Our data further suggest that corticosteroid treatment in COVID-19 is a risk factor for acquiring secondary bacterial infections in the ICU.

The differing risk of ICU-AI in patients with COVID-19 as opposed to influenza accords with other studies^2,11,21–24^. It may be explained by factors such as increased demand on the healthcare system during the COVID-19 pandemic^11,25^, alterations of immune responses caused by SARS-CoV-2^21^, a high proportion of ARDS in COVID-19, more frequent prone positioning^23^, and prolonged IMV and ICU stays^11,26^. Although we noted no difference in ICU LoS between the COVID-19 and influenza cohorts, there was a small difference in time on IMV. Consistent with findings from other studies^23,26^, more males were observed in critical COVID-19 cases than in influenza cases. This may account for the different incidence rates, as this and other studies suggest that male gender is a risk factor for ICU-AI^15,27^.

There was no significant difference in the percentage of patients with corticosteroid treatment between the two cohorts. However, the indication for corticosteroid treatment to patients with influenza was airway obstruction and/or sepsis with lower doses and shorter duration than recommended in severe COVID-19. Furthermore, antibiotic treatment on admission has been shown to be a risk factor for ICU-AI^2,28,29^, and early initiation of antibiotics was high throughout the pandemic, despite the low frequency of co-infections on admission in patients with COVID-19. On the other hand, it is possible that the lower incidence of ICU-AI in the influenza cohort is partly explained by earlier diagnosis and targeted treatment of co-infection, while some co-infections in the COVID-19 cohort might been missed initially and later misinterpreted as ICU-AI.

As the pandemic developed, incidence rates of ICU-AI in patients with COVID-19 increased. A similar pattern, but with slightly lower incidence rates, was seen in a recent Swedish study on VA-LRTI^29^. The differing incidence rates of ICU-AI during the pandemic can be partly explained by a shift in corticosteroid treatment, for as our study and several others have suggested, corticosteroid treatment is a risk factor for ICU-AI^2,15,22,29,30^. Moreover, later in the pandemic patients were more critically ill and had more co-infections on admission, possibly affecting the risk of ICU-AI. Nor can we rule out other variables, such as changes in management or staffing at the ICU^31^, different SARS-CoV-2 strains, or vaccinations^32^, any of which may have affected the risk of ICU-AI throughout the pandemic.

Other studies have demonstrated the same association between ICU LoS and IMV duration, while reports on mortality are conflicting^15,24,29,30,33^. Our findings demonstrate an increased risk of mortality with ICU-AI in patients with corticosteroid treatment as compared to patients who have not received corticosteroids. This may in part reflect the higher mortality that occurred in later waves in contrast to the first. Although glucocorticoids have been shown to reduce mortality^12,34^, later studies have indicated that not all patients with severe COVID-19 may benefit from corticosteroid treatment^15,35,36^. We did not find any interaction between age and corticosteroid treatment on the risk of ICU-AI, but it cannot be ruled out that certain patient categories might be affected differently by corticosteroid treatment. Further risk–benefit studies of the association between corticosteroid treatment, ICU-AI, and outcome in hospitalized patients are needed.

The microbial pattern we observed in VA-LRTI is consistent with that seen elsewhere^11,14,22,29,30^. Although we found a larger discrepancy between the two cohorts than other studies observed^11,23,24,37^, this may have been due to the small number of patients with influenza and ICU-AI. A shift in the microbial pattern was observed between early and late VA-LRTI, with an increase in more difficult-to-treat microbes in later stages, consistent with findings reported in other studies^11,29,30^. Possible explanations for this are alterations in lung microbiota^38^, increase of biofilm-active bacteria^39^, as well as an overuse of antibiotics^2^. We noted a change throughout the pandemic towards more broad-spectrum antibiotic treatment on admission in patients with COVID-19. Broad-spectrum antibiotics are a risk factor for ICU-AI^28^ and may possibly facilitate the development of more complicated infections. Although the rate of MDRO was comparatively low^23,40^, there is a risk of decreasing antibiotic susceptibility with the overuse of antibiotics^41,42^.

The major strengths of our study are the large sample size of patients on IMV due to COVID-19 and our detailed examination of the medical charts for each case. There are however some important limitations to consider: First, the retrospective nature of the study. Second, the small comparison group, due to the relatively few patients on IMV as a result of influenza, especially during the COVID-19 pandemic. The inclusion period for the two cohorts also differed somewhat, possibly affecting the prevalence of MDRO. Third, most patients receiving corticosteroid treatment were hospitalized after the first wave, so it is possible that there were coinciding changes in management that further affected the risk of ICU-AI. Fourth, most samples from the lower respiratory tract were not taken with protected brush. This may have resulted in some colonization cultures and contaminations being included for analysis.

## Conclusion

Secondary infections among ICU patients with COVID-19 are a common complication associated with a more complex course of disease. Their high incidence rates during the COVID-19 pandemic may partly be due to frequent corticosteroid treatment. Given the increased use of corticosteroids for severe viral and bacterial pneumonia, their impact on ICU-AI merits further evaluation.

### Supplementary Information

Supplementary Information.

## Data Availability

The datasets used and/or analyzed during the current study are available from the corresponding author on reasonable request.

## Abbreviations

ARDS: Acute respiratory distress syndrome
BAL: Bronchoalveolar lavage
BSI: Blood stream infection
CCI: Charlson Comorbidity Index score
CI: Confidence intervals
ICD-10: International Classification of Diseases 10th Revision
ICU: Intensive care unit
ICU-AI: Intensive care unit-acquired infection
IL6: Interleukin 6
IMV: Invasive mechanical ventilation
JAK: Janus Kinase
LoS: Length of stay
MDRO: Multidrug-resistant organisms
PCR: Polymerase chain reaction
SAPS 3: Simplified acute physiology score III
SU: Sahlgrenska University Hospital
VA-LRTI: Ventilator-associated lower respiratory tract infection
VAP: Ventilator-associated pneumonia
