# Supplementary material for: Intensive care unit-acquired infections more common in patients with COVID-19 than with influenza
Source: Sci Rep. 2024 Jul 19;14:16655. doi: 10.1038/s41598-024-67733-z (PMC11271526; doi:10.1038/s41598-024-67733-z)
Supplement: Supplementary file 1 — Supplementary Information. [file 41598_2024_67733_MOESM1_ESM.pdf]

Intensive Care Unit-Acquired Infections More Common in Patients with COVID-19 than with Influenza

Josefine Beck-Friis*,[1,2] Magnus Gisslén,[1,2,3] Staffan Nilsson[4], Anna Lindblom,[1,5] Jonatan Oras,[6,7] Aylin Yilmaz[1,2]

[1]Department of Infectious Diseases, Institute of Biomedicine, Sahlgrenska Academy, University of Gothenburg, Gothenburg, Sweden
[2]Region Västra Götaland, Department of Infectious Diseases, Sahlgrenska University Hospital, Gothenburg, Sweden
[3]Public Health Agency of Sweden, Solna
[4]Department of Laboratory Medicine, Institute of Biomedicine, Sahlgrenska Academy, University of Gothenburg, Gothenburg, Sweden
[5]Region Västra Götaland, Department of Clinical Microbiology, Sahlgrenska University Hospital, Gothenburg, Sweden
[6]Department of Anesthesiology and Intensive Care Medicine, Institute of Clinical Sciences, Sahlgrenska Academy, University of Gothenburg, Gothenburg, Sweden
[7]Region Västra Götaland, Department of Anesthesia and Intensive Care, Sahlgrenska University Hospital, Gothenburg, Sweden

*Corresponding author
Department of Infectious Diseases
Sahlgrenska University Hospital
SE-416 85 Gothenburg
Sweden

Email: Josefine.Beck-Friis@gu.se

Supplementary Material

List of Content

Page 3. Supplementary Table 1. List of ICD-10 Codes.

Page 4. Supplementary Table 2. Subgroup Analysis COVID-19 Cohort with Comparison wave 1 – wave 4

Page 5. Supplementary Table 3. Subgroup Analysis of Patients on Invasive Mechanical Ventilation due to Influenza.

Page 6. Supplementary Table 4. COVID 19 Cohort Subgroup Analysis of the Microbiological Findings in Ventilator Associated Lower Respiratory Tract Infections.

Page 7. Supplementary Table 5. Microbial Cultures from Patients in Ventilator due to COVID-19 and Influenza.

Page 8. Supplementary Figure 1.

Supplementary Table 1. List of ICD-10 Codes

ICD-10 Code	Description
U07.1	COVID-19, virus identified
J10.0	Influenza with pneumonia, seasonal influenza virus identified
J10.1	Influenza with other respiratory manifestations, seasonal influenza virus identified
J10.8	Influenza with other manifestations, seasonal influenza virus identified

Summary of codes used for inclusion in the study. Abbreviations: ICD-10, International Classification of Diagnosis 10th Revision.

Supplementary Table 2. Subgroup Analysis of the COVID-19 Cohort with Comparison wave 1 – wave 4

	**Wave 1 (N = 160)**	**Wave 2 (N = 112)**	**Wave 3 (N = 144)**	**Wave 4 (N = 20)**	***P* value**
Median (range) or n (%)					
ICU-AI	56 (36)	58 (52)	72 (50)	6 (30)	.008
Days at ICU until first ICU-AI	11 (3–46)	8 (2–55)	9 (3–56)	7 (3–26)	.24
Incidence rate ICU-AI per[a] 1000 days (95% CI)	23.7 (17.9–30.8)	8.6 (29.3–50.0)	35.7 (27.9–44.9)	30.6 (11.2–67.0)	.041
VA-LRTI	38 (24)	45 (40)	60 (42)	6 (30)	.004
Days on IMV until first VA-LRTI	10 (2–35)	7 (1–53)	8 (1–56)	6 (1–25)	.25
Incidence rate VA-LRTI per 1000 days (95% CI)	17.1 (12.1–23.6)	30.6 (22.3–41.0)	30.4 (23.2–39.1)	40.3 (14.8–87.7)	.010
Age in years	60 (22–90)	67 (20–86)	64 (21–90)	61 (31–84)	.015
Female sex	30 (19)	26 (23)	44 (31)	7 (35)	.072
Diabetes	40 (25)	39 (35)	38 (26)	8 (40)	.19
Number comorbidities	2 (0–5)	2 (0–6)	1 (0–5)	2 (0–5)	.33
Charlson Comorbidity Index score	2 (0–9)	3 (0–9)	2 (0–9)	4 (0–6)	< .001
Immunotherapy baseline	8 (5)	9 (8)	9 (6)	4 (20)	.077
SAPS 3[b]	49 (34–77)	54 (34–100)	52 (37–91)	56 (37–108)	.006
Co-infection	12 (8)	11 (10)	21 (15)	5 (25)	.049
Antibiotic treatment within 48h	145 (91)	103 (92)	134 (93)	17 (85)	.63
First antibiotic administered					
Cefotaxime	137 (86)	89 (79)	109 (76)	10 (50)	.002
Piperacillin/tazobactam	16 (10)	17 (15)	28 (19)	9 (45)	< .001
Corticosteroid treatment	33 (21)[c]	106 (95)	141 (98)	18 (90)	< .001
Days in ICU	17 (4–103)	20 (2–62)	21 (4–81)	11 (3–45)	.004
Days on IMV	15 (3–92)	16 (2–61)	19 (3–77)	9 (2–36)	.006
30-day mortality	32 (20)	28 (25)	34 (24)	8 (40)	.23
90-day mortality	40 (25)	38 (34)	44 (31)	8 (40)	.30

Abbreviations: ICU, intensive care unit; ICU-AI, ICU-acquired infection; IMV, Invasive Mechanical Ventilation; n/a, not applicable; SAPS 3, Simplified Acute Physiology Score 3 to predict hospital mortality on ICU admission; VA-LRTI, ventilator associated lower respiratory tract infections; 95% CI, Poisson 95% confidence interval.

Definitions: Wave 1, 1 Feb 2020–27 Sep 2020; Wave 2, 28 Sep 2020–31 Jan 2021; Wave 3, 1 Feb 2021–7 Nov 2021; Wave 4, 8 Nov 2021–31 Mar 2022; Co-infection, bacterial infection diagnosed < 48 hours after admittance to Intensive Care Unit (ICU) due to SARS CoV-2 or influenza virus infection.

P value < .05 means that there is no statistical difference between the groups.

[a]Times at risk for patients with data missing (three from wave 1, two from wave 2) were estimated to half their lengths of stay in ICU

[b]n=433 due to data missing

[c]n=157 due to data missing

Supplementary Table 3. Subgroup Analysis of Patients on Invasive Mechanical Ventilation due to Influenza.

	Total (N = 44)	ICU-AI (N = 7)	No ICU-AI (N = 37)	*P* value
Median (range) or n (%)				
Female sex	19 (43)	2 (29)	17 (46)	.68
Age; in years	63 (29–86)	63 (41–72)	62 (29–86)	.77
Charlson Comorbidity Index score	2 (0–7)	1 (0–6)	3 (0–7)	.35
Number of comorbidities	2 (1–7)	1 (0–4)	2 (0–7)	.021
Immunosuppressive therapy baseline	4 (9)	0	4 (11)	1.00
SAPS 3	60 (40–93)	58 (43–60)	61 (40–93)	.16
Corticosteroid treatment at ICU	24 (55)	5 (71)	19 (51)	.43
Number of antibiotic drugs	3 (1–15)	6 (2–15)	3 (1–10)	.006
Days in ICU	19 (3–34)	40 (8–74)	16 (3–34)	.007
Days on IMV	13 (3–68)	36 (8–68)	8 (3–32)	.003
30-day mortality	8 (18)	2 (29)	6 (16)	.59
90-day mortality	9 (20)	2 (29)	7 (19)	.62

Abbreviations: ICU, Intensive Care Unit; ICU-AI, ICU-Acquired Infection; IMV, Invasive Mechanical Ventilation; SAPS 3, Simplified Acute Physiology Score 3 to predict hospital mortality on ICU admission.

Supplementary Table 4. COVID 19 Cohort Subgroup Analysis of the microbiological findings in Ventilator Associated Lower Respiratory Tract Infections

	All VA-LRTI (N = 210)	Early VA-LRTI (N = 102)	Late VA-LRTI (N = 108)
n (%)			
Gram-positive bacteria	71 (34)	45 (44)	26 (22)
Staphylococcus aureus	54 (26)	33 (32)	21 (19)
Enterococcus faecalis	9 (4)	6 (6)	3 (3)
Other gram-positive bacteria[a]	8 (4)	6 (6)	2 (2)
MDRO (% of gram-positive bacteria)	6 (8)	4 (9)	2 (8)
Gram-negative bacteria	138 (66)	57 (56)	81 (75)
Klebsiella spp	42 20)	20 (20)	22 (20)
Escherichia coli	19 (9)	9 (9)	10 (9)
Pseudomonas aeruginosa	23 (11)	7 (7)	16 (15)
Serratia marcescens	7 (3)	2 (2)	5 (5)
Stenotrophomonas maltophila	13 (6)	1 (1)	12 (11)
Enterobacter spp	11 (5)	6 (6)	5 (5)
Other gram-negative bacteria[b]	23 (11)	12 (12)	11 (10)
MDRO (% of gram-negativa bacteria)	20 (15)	9 (16)	11 (14)
Aspergillus fumigatus	1 (0.5)	0	1 (1)
MDRO[c]	26 (12)	13 (13)	13 (12)

Abbreviations: MDRO, Multi-drug resistant organisms; VA-LRTI, Ventilator-Associated Lower Respiratory Tract Infection

Descriptive data on bacterial and fungal isolates associated with VA-LRTI in patients on invasive mechanical ventilation due to COVID-19 presented in numbers and percentages (%).

Definitions: early VA-LRTI, VA-LRTI day 0-9 on invasive mechanical ventilation; late VA-LRTI, VA-LRTI > 9 days on invasive mechanical ventilation. The cut-off at nine days was set according to the median time until first intensive care unit-acquired infection in this cohort.

[a]Early VA-LRTI: 2 *Enterococcus faecium*, 3 *Streptococcus pneumoniae,* 1 *β-haemolytic Streptococcus* spp; late VA-LRTI: 1 *Enterococcus faecium,* 1 *Granulicatella adiacens*

[b]Early VA-LRTI: 4 *Haemophilus* spp, 3 *Proteus mirabilis,* 1 *Pantoea* spp, 2 *Acinetobacter baumanii,* 1 *Chryseobacterium* spp, 1 *Elizabethkingia meningoseptica*; late VA-LRTI: 2 *Haemophilus* spp, 4 *Citrobacter* spp, 1 *Sphingomonas* spp, 2 *Acinetobacter baumanii*, 1 *Achromobacter* spp, 1 *Morganella morganii*

[c]8 *Pseudomonas aeruginosa,* 6 *Escherichia coli,* 4 *Klebsiella* spp, 2 *Enterobacter* spp, 6 *Staphylococcus aureus* (MRSA)

Supplementary Table 5. Microbial Cultures from Patients on Invasive Mechanical Ventilation due to COVID-19 and Influenza

	COVID-19 (N = 436)	Influenza (N = 44)
Median (range) or n (%)		
Blood cultures	3930	279
Blood cultures per patient	8 (0–53)	5 (0–27)
Positive blood cultures	613 (16)	36 (6)
Positive blood cultures per patient	1 (0–14)	0 (0–4)
All pathogens cultured in blood	703	43
Significant pathogens in blood	208 (30)	4 (9)
Lower respiratory tract cultures	1837	103
Lower respiratory tract cultures per patient	3 (0–28)	2 (0–13)
Positive lower respiratory tract cultures	651 (35)	13 (2)
Pathogens cultured lower respiratory tract	765	14
Significant pathogens in lower respiratory tract	539 (11)	13 (93)

A descriptive presentation of the bacterial and fungal cultures taken between 48 hours after admittance to the intensive care unit and 48 hours after discharge from the intensive care unit. No candida has been included in the analysis of cultures from the lower respiratory tract. Categorical data are presented as numbers and percentages (%) where applicable, and continuous data are presented as median and range (minimum–maximum).

Supplementary Figure 1.

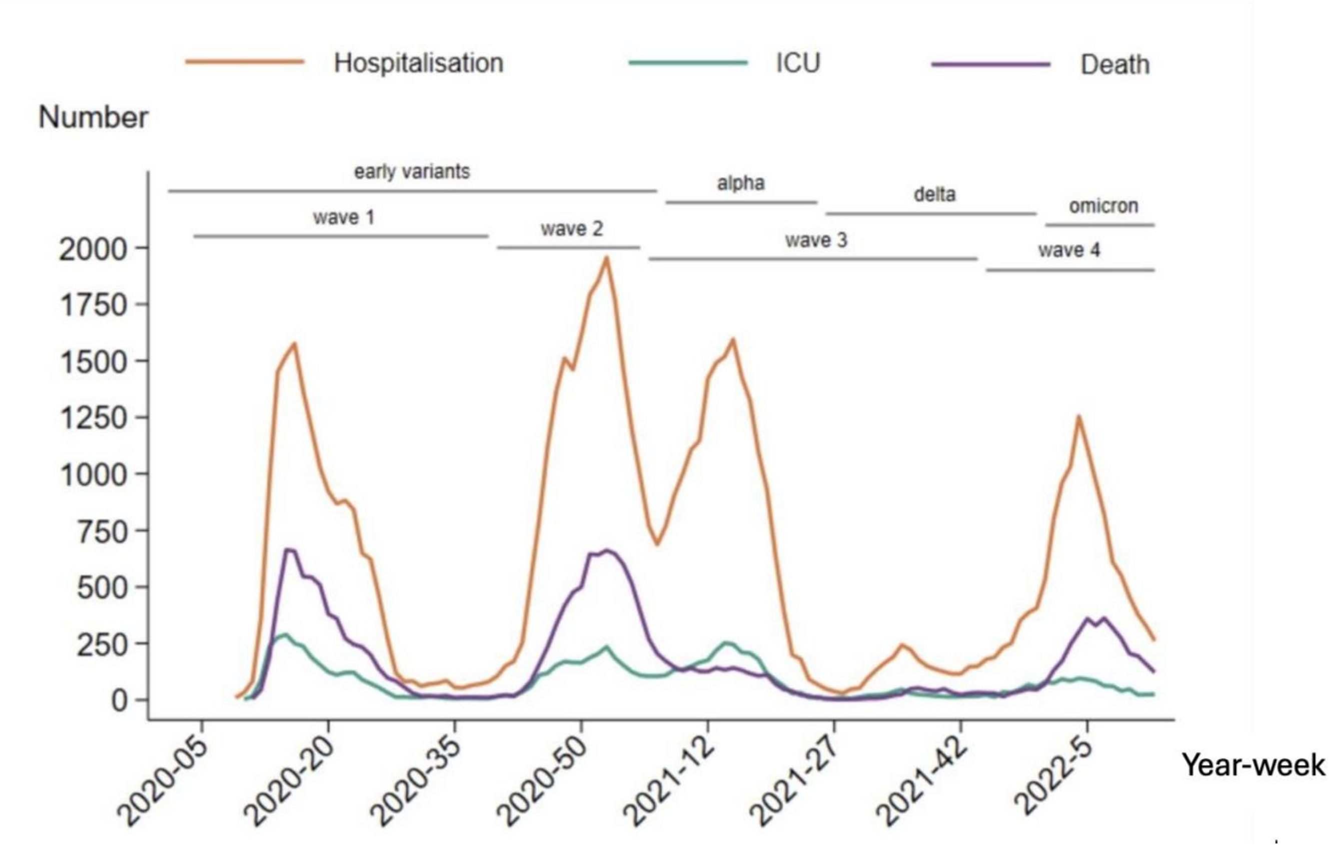

Supplementary Figure 1. Graph showing the variations in COVID-19 related hospitalization, ICU admissions, mortality, and variants of SARS-CoV-2 in Sweden during the COVID-19 pandemic. The waves are here determined by the variations in hospitalization at Sahlgrenska University Hospital in Gothenburg, Sweden. Abbreviations: ICU, Intensive care unit. Sources: Public Health Agency of Sweden, Smi-Net – National Covid-19 notifiable disease reporting, SIR – Swedish Intensive Care Register, National Patient Register, Swedish Tax Agency.
